# Riociguat Alleviates Cisplatin-Caused Kidney Injury by Suppressing Oxidative Stress and Inflammation

**DOI:** 10.3390/biology14101346

**Published:** 2025-10-02

**Authors:** Yousuf M. Al Suleimani, Yousra Nomeir, Raya Al Maskari, Haytham Ali, Priyadarsini Manoj, Aly M. Abdelrahman

**Affiliations:** 1 Department of Pharmacology and Clinical Pharmacy, College of Medicine and Health Sciences, Sultan Qaboos University, P.O. Box 35, Muscat 123, Oman; y.nomeir@squ.edu.om (Y.N.); r.maskari@squ.edu.om (R.A.M.); priyadarsinimanoj@gmail.com (P.M.); abdelrahman@squ.edu.om (A.M.A.); 2 Department of Animal and Veterinary Sciences, College of Agricultural and Marine Sciences, Sultan Qaboos University, P.O. Box 35, Muscat 123, Oman; h.ali@squ.edu.om

**Keywords:** cisplatin, acute kidney injury, riociguat, antioxidation, anti-inflammation

## Abstract

Background: Cisplatin (CP), a platinum-based chemotherapeutic agent, is widely used to treat cancer but causes nephrotoxicity. Riociguat, a soluble guanylate cyclase (sGC) stimulator that enhances the nitric oxide–sGC–cGMP signaling pathway, was investigated for its potential protective effects against cisplatin-induced nephrotoxicity. Materials and Methods: Rats were randomly divided into four equal groups (six rats each) and treated for nine consecutive days. The first and second groups were given oral carboxymethylcellulose 0.5% (vehicle) for 9 days, and on day 6 were injected intraperitoneally with saline or CP, respectively. The third and fourth groups were treated orally with two doses of riociguat (3 and 10 mg/kg/day) for 9 days, and received intraperitoneal injections of CP on day 6. Blood, urine, and kidney tissues were analyzed 24 h after the last treatment. Results: CP significantly elevated the markers of kidney function, including uric acid, serum creatinine, and urea. CP also caused histological kidney damage. Antioxidant markers, including catalase (CAT), glutathione reductase (GR), superoxide dismutase (SOD), and total antioxidant capacity (TAC) were significantly reduced, while inflammatory cytokines (IL-1β, IL-6, and TNF-α) and lipid peroxidation (MDA) were markedly increased. Riociguat improved kidney structure and significantly reduced kidney function markers, MDA, and inflammatory cytokines while restoring GR, TAC, SOD, and CAT activities. Conclusions: These results indicate that riociguat exerts protection of the kidneys from CP-caused kidney damage by antioxidation and anti-inflammation. Riociguat may have potential as an adjunct therapy to mitigate CP-associated nephrotoxicity.

## Simple Summary

Cisplatin is a medicine used to treat many types of cancer, although one of its major drawbacks is that it can cause serious damage to the kidneys. In this study, we tested whether another drug, riociguat, could protect the kidneys from this harmful effect. Using a rat model, we found that treatment with riociguat improved kidney function in animals that received cisplatin. Riociguat reduced the build-up of harmful substances in the blood, lowered markers of inflammation, and restored the natural defense systems that protect kidney cells from damage. The drug also improved the structure of kidney tissue that had been harmed by cisplatin. These results suggest that riociguat helps the kidneys by reducing both inflammation and oxidative stress, which are key drivers of injury. Our findings highlight the potential of riociguat as an additional therapy to reduce kidney damage in cancer patients receiving cisplatin. While these results are promising, further studies in humans are needed to confirm whether this protective effect can be safely applied in clinical practice.

## 1. Introduction

Acute kidney injury (AKI) is a serious medical condition characterized by an abrupt decline in renal function, leading to impaired elimination of waste products, disturbances in fluid balance, and electrolyte dysregulation, typically reflected by elevated serum creatinine and blood urea nitrogen levels. AKI is associated with increased morbidity and mortality and may progress to chronic or end-stage renal failure in survivors [1,2]. The Kidney Disease: Improving Global Outcomes (KDIGO) classification provides a standardized clinical definition and management framework for AKI based on changes in serum creatinine and urine output. According to KDIGO, AKI is diagnosed when serum creatinine increases by ≥0.3 mg/dL within 48 h, rises to ≥1.5 times the baseline within 7 days, or when urine output falls below 0.5 mL/kg/h for at least 6 h. The severity of AKI is staged from 1 to 3 according to the magnitude of these changes. Use of the KDIGO classification allows for consistent assessment of AKI severity and outcomes in both clinical practice and experimental studies [3].

AKI is linked to higher rates of morbidity and mortality and frequently advances to chronic kidney disease (CKD) or end-stage renal disease (ESRD) in survivors. The pathophysiology of AKI includes many factors, such as decreased renal perfusion, endothelial dysfunction, inflammatory infiltration, oxidative stress, microthrombi, direct tubular injury, obstruction, and cytokine-induced injury [4]. Oxidative damage—leading to changes in DNA and RNA, the peroxidation of lipids, or changes in the structure of proteins—is an important cause of kidney damage in AKI [5].

Cisplatin (CP), a widely used chemotherapeutic agent, is effective in treating various cancers such as lung, ovarian, and bladder cancer [6]; however, its clinical use is often restricted due to nephrotoxicity, a serious side effect that affects 30–40% of patients receiving cisplatin-based therapies [7]. CP accumulates in renal tubular cells, triggering activation of inflammatory pathways, excessive production of reactive oxygen species (ROS), and mitochondrial damage [8,9]. These processes lead to structural and functional impairments in the kidneys, evidenced by increased levels of serum creatinine, urea, and other markers of kidney damage [10]. While current protective measures, such as hydration and antioxidant therapy, help reduce the risk of nephrotoxicity, they are insufficient in many cases, necessitating the exploration of novel therapeutic approaches [11].

Rodent models of CP-caused AKI are widely used to investigate the mechanisms of renal injury and to test potential therapeutic interventions. These models closely mimic the pathological features of human AKI, including tubular damage, oxidative stress, and inflammation. They are invaluable tools for understanding the molecular underpinnings of AKI and evaluating strategies to mitigate CP-induced nephrotoxicity [12].

Riociguat, a pioneering soluble guanylate cyclase (sGC) stimulator, is approved for treating pulmonary arterial hypertension (PAH) and chronic thromboembolic pulmonary hypertension (CTEPH) [13,14]. By enhancing the nitric oxide (NO)-sGC-cyclic guanosine monophosphate (cGMP) pathway, riociguat improves vascular relaxation, reduces vascular resistance, and promotes endothelial function. These mechanisms suggest a potential protective effect against oxidative stress and inflammation, key drivers of AKI. Although primarily studied in cardiovascular diseases, riociguat’s ability to modulate these pathways offers a promising avenue for addressing renal dysfunction; however, its effects on kidney injury, particularly cisplatin-induced AKI, remain largely unexplored [15]. This work aimed to explore the potential renoprotective effects of riociguat in a rat model of CP-induced AKI. The impact of riociguat on renal function, histological changes, oxidative stress, and inflammatory markers was explored. This study aims to offer insights into the therapeutic potential of riociguat in reducing CP-induced nephrotoxicity and advancing AKI management strategies.

## 2. Materials and Methods

### 2.1. Drugs, Chemicals, and Biochemical Analysis

CP was obtained from the University Medical City Pharmacy, Oman, while riociguat was purchased from the Chinese company ZhiShang Chemical, Jinan. Kidney function markers, including albumin, urea, uric acid, and creatinine, were measured using a Mindray BS-120 automated chemistry analyzer (Mindray Bio-Medical Electronics Co., Shenzhen, China). Osmolality was determined using the freezing-point depression method with an Osmomat 3000 Osmometer (Gonotec GmbH, Berlin, Germany).

### 2.2. Animals

Rats (male, Wistar), ranging in weight from 200 to 300 g, were provided by the Small Animal House facility at Sultan Qaboos University, Oman. The animals were kept in polypropylene cages maintained at 22 ± 2 °C, with a 12 h light–dark cycle starting at 6:00 AM, at approximately 60% humidity. The rats had unrestricted access to standard laboratory chow (Oman Mills, Muscat, Oman) and tap water. This study received ethical clearance from the University Ethical Committee for Animal Use in Research at Sultan Qaboos University, Oman (SQU/EC-AUR/2021-2022-13). All experimental procedures followed international guidelines for animal care, including the European Directive 2010/63/EU and the NIH *Guide for the Care and Use of Laboratory Animals* (8th edition, 2011). Both statistical and ethical aspects were considered in determining the number of rats per group. A sample size of n = 6 per group is consistent with the recommendations of the British Journal of Pharmacology guidelines, which suggest this as an appropriate minimum for detecting biologically relevant effects in preclinical pharmacology studies. During the experiments, the rats were treated in such a way as to minimize the pain, suffering, distress, or lasting harm.

### 2.3. Animal Grouping

Following a one-week acclimation period, 24 rats were arbitrarily grouped into four groups (n = 6) and treated for a period of nine days, as follows:

Group 1 (Control): Administered an oral vehicle (0.5% carboxymethylcellulose) for nine days, and an i.p. injection of saline on day 6.

Group 2 (CP only): Given an oral vehicle daily for nine days, and CP (i.p., 6 mg/kg) was administered on day 6.

Group 3 (Cisplatin + Riociguat 3 mg/kg): Treated with oral riociguat at the stated dose for nine days, and CP (i.p., 6 mg/kg) was administered on day 6.

Group 4 (Cisplatin + Riociguat 10 mg/kg): Treated with oral riociguat at the stated dose for nine days, and CP (i.p., 6 mg/kg) was administered on day 6.

Riociguat was dissolved in 0.5% carboxymethylcellulose to ensure proper solubility and oral administration. Accordingly, the sham group received the same vehicle to serve as a control. Riociguat did not adequately dissolve in water or other available solvents, whereas 0.5% carboxylmethylcellulose provided complete solubilization. Riociguat or the vehicle was administered orally by gastric gavage using an appropriately sized feeding needle. The trained technician carefully observed each rat during and after administration to ensure that the full dose was delivered, and no regurgitation or expulsion of the administered solution was noted.

The doses of cisplatin and riociguat were selected according to previously published work [16,17].

### 2.4. Experimental Protocol

Following the treatment period, the rats were placed in metabolic cages to facilitate the collection of urine over 24 h. Final-day body weight was taken on the 10th day. Afterwards, anesthesia was administered via intraperitoneal injection of ketamine (75 mg/kg) and xylazine (5 mg/kg). Blood samples (4–5 mL) were drawn from the abdominal aorta and processed through centrifugation at 900× *g* for 15 min at 4 °C to separate the plasma. Both urine and plasma samples were preserved at −80 °C for future examinations. Euthanasia was carried out using an excessive dose of ketamine and xylazine. The kidneys were then excised, blotted to remove excess moisture, and weighed. A section of the right kidney was preserved in formalin for histopathological studies, whereas the remaining tissue was quickly wrapped in aluminum foil, flash-frozen using liquid nitrogen, and stored at −80 °C for biochemical evaluations.

### 2.5. Preparation of Tissue Homogenates

Kidney tissue was processed by homogenizing it in a phosphate buffer (pH 7.4) supplemented with a protease inhibitor (1 µg/mL). The resulting homogenate underwent centrifugation at 800× *g* for five minutes at 4 °C, and the supernatant (S1) was extracted for TAC and MDA assessments. The remaining fraction was subjected to an additional centrifugation step at 10,500× *g* for 15 min at 4 °C, yielding a post-mitochondrial supernatant (PMS) designated for evaluating SOD, GR, and CAT activity [18].

### 2.6. Kidney Function Markers

Urine albumin and serum levels of urea, creatinine, and uric acid were quantified by the Mindray BS 120 fully automated chemistry analyzer from Shenzhen Mindray Bio-Medical Electronics Co. (Shenzhen, China) using Mindray kits.

### 2.7. Oxidative Stress Parameters

Kidney oxidative stress markers were assessed using commercially available kits. SOD and GR were quantified by colorimetric methods with BioVision kits (Milpitas, CA, USA). MDA and TAC were evaluated using My BioSource kits (San Diego, CA, USA).

### 2.8. Inflammatory Markers

Levels of inflammatory markers such as IL-1β, TNF-α, IL-6, NGAL, and NAG were quantified using ELISA kits sourced from Thermo Fisher Scientific (Waltham, MA, USA) and Cusabio Biotech (Wuhan, China). Kidney supernatants were prepared following homogenization and centrifugation, and samples were added to 96-well plates pre-coated with specific antibodies.

### 2.9. Histopathological Analysis

The kidneys were weighed and preserved in 10% neutral-buffered formalin for 24 to 48 h. After fixation, they underwent dehydration using a graded ethanol series, followed by clearing with xylene and embedding in paraffin. Tissue sections, each four microns thick, were prepared and stained with hematoxylin and eosin (H&E) or Sirius Red to evaluate fibrosis. Microscopic analysis was then performed. Blinded scoring was performed by a pathologist following established protocols [19]. Briefly, renal tubular necrosis was assessed using a scoring method on a scale of 0–4, where 0 = normal, no necrosis; 1 < 10%; 2 = 10–25%; 3 = 26–75%; 4 > 75%. Three 40X microscopic fields were analyzed from each kidney section of each animal in the 4 groups, and the score was calculated according to the mean percentage.

### 2.10. Statistical Analysis

The data are expressed as mean ± SEM and were statistically analyzed using one-way ANOVA, followed by Bonferroni’s post hoc test in GraphPad Prism (version 5.03, San Diego, CA, USA). A *p*-value of less than 0.05 was considered statistically significant.

## 3. Results

### 3.1. Effect on Physiological Parameters

As presented in Table 1, baseline and final body weights did not differ significantly among the four groups. However, CP treatment significantly reduced the percentage change in body weight compared to the control group, and riociguat at both doses (3 and 10 mg/kg) failed to counteract this effect. CP administration led to a significant increase in relative kidney weight, which was reversed by high-dose riociguat (10 mg/kg). Water intake was significantly higher in rats treated with CP alone or in combination with riociguat at either dose compared to the control group. Additionally, urine output and osmolality were elevated in all treatment groups relative to control, with only the high dose of riociguat (10 mg/kg) mitigating CP’s effects on both parameters.

### 3.2. Effect on the Renal Biomarkers

**Figure 1:**
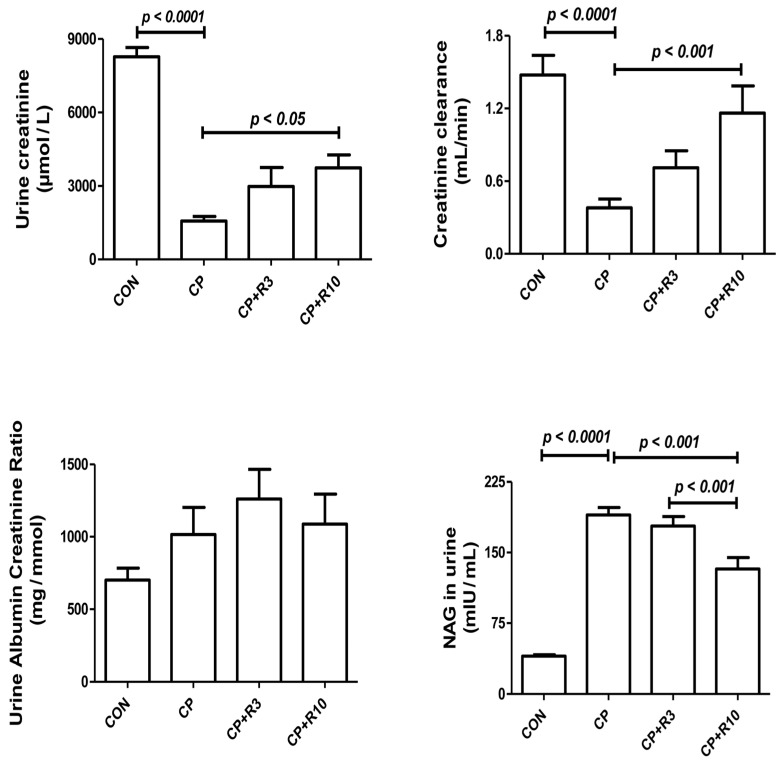
Effect of riociguat (R) on urinary parameters in rats with cisplatin (CP)-induced acute kidney injury (AKI). The figure presents the urine albumin-to-creatinine ratio, creatinine, creatinine clearance, and N-acetyl-β-D-glucosaminidase (NAG) in control rats, as well as in rats treated with cisplatin (CP) alone or in combination with riociguat at 3 mg/kg (R3) or 10 mg/kg (R10). Each bar represents the mean ± SEM (n = 6). Group differences were analyzed using one-way ANOVA followed by Bonferroni’s multiple comparison test, with significance set at *p* < 0.05.

Table 2 shows that plasma levels of kidney function markers, including urea, creatinine, uric acid, and NGAL, were significantly elevated in the CP group compared to the control group. Treatment with riociguat (3 and 10 mg/kg) mitigated these CP-induced changes. Figure 1 shows that CP significantly reduced the levels of urine creatinine, as well as creatinine clearance, effects that were mitigated by riociguat at the higher dose (10 mg/kg). Furthermore, both doses of riociguat significantly reversed CP-induced decreases in NAG.

### 3.3. Effect on Biomarkers of Oxidative Stress

**Figure 2:**
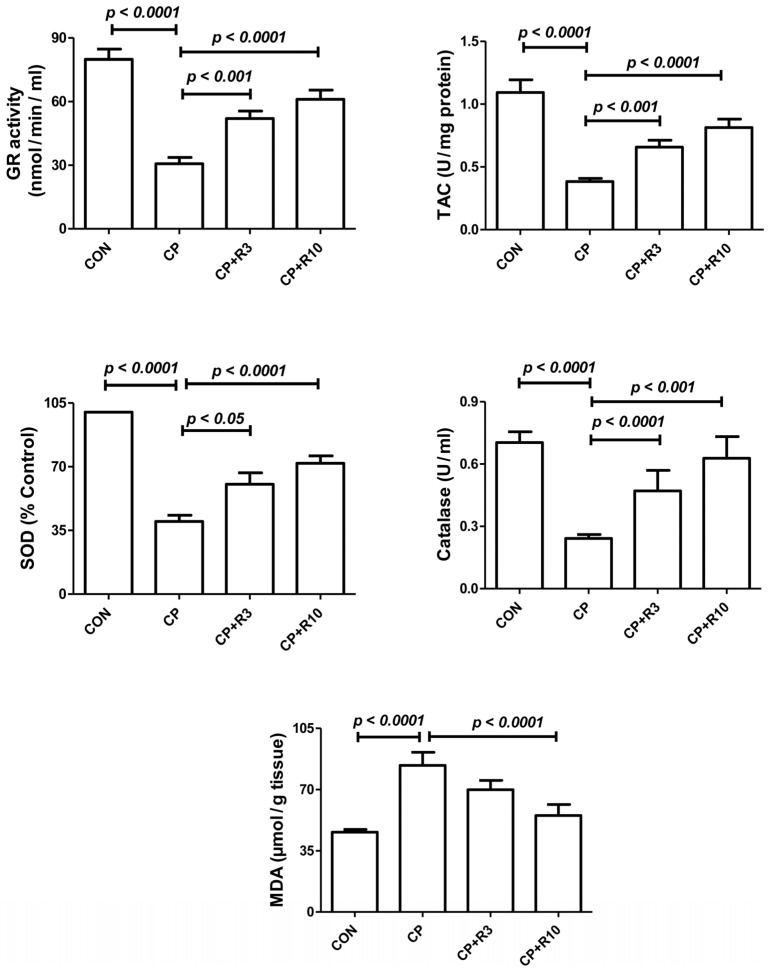
Renal levels of superoxide dismutase (SOD), catalase (CAT), total antioxidant capacity (TAC), glutathione reductase (GR), and malondialdehyde (MDA) in control rats, and rats treated with CP alone or in combination with riociguat at 3 mg/kg (R3) or 10 mg/kg (R10). Data are presented as mean ± SEM (n = 6). Group differences were analyzed using one-way ANOVA followed by Bonferroni’s multiple comparison test, with significance set at *p* < 0.05.

The kidney tissue homogenates of the control, CP, and riociguat-treated groups exhibited varying activities of antioxidant enzymes (TAC, SOD, GR, and CAT), as shown in Figure 2. Antioxidant enzyme activity was significantly lower in the CP group compared to the control group. Treatment with riociguat at both doses (3 and 10 mg/kg) mitigated these CP-induced reductions. Malondialdehyde (MDA) is a widely studied marker of lipid peroxidation (LPO). Figure 2 also presents LPO levels in the renal tissue homogenates of the control, CP, and riociguat-treated groups. LPO levels were significantly elevated in the CP group compared to the control group, an effect that was significantly reversed by riociguat at 10 mg/kg.

### 3.4. Effect on Inflammatory Cytokines

**Figure 3:**
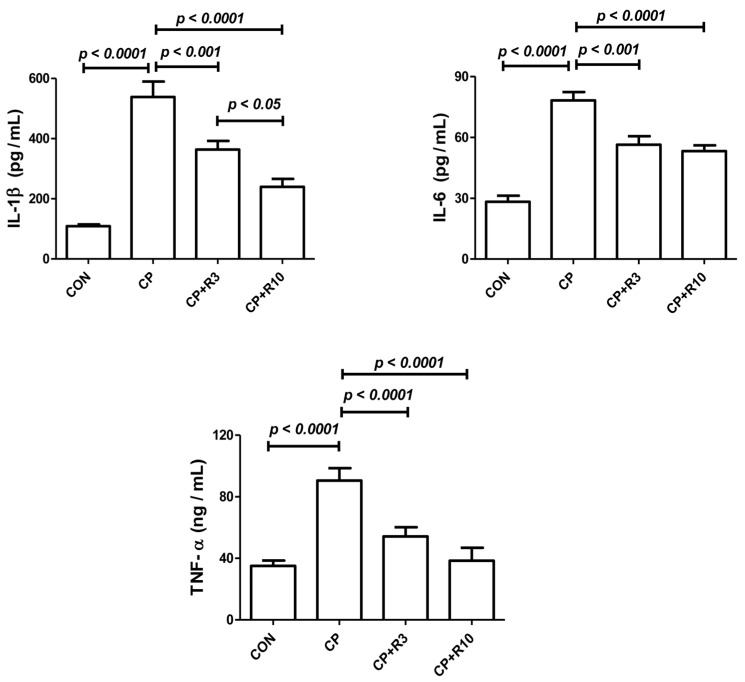
Plasma concentrations of tumor necrosis factor-alpha (TNF-α), interleukin-1 beta (IL-1β), and interleukin-6 (IL-6) in control rats and those treated with CP alone or in combination with riociguat (3 mg/kg, R3; 10 mg/kg, R10). Data are expressed as mean ± SEM (n = 6). Statistical analysis was performed using one-way ANOVA followed by Bonferroni’s multiple comparison test, with significance set at *p* < 0.05.

Figure 3 illustrates the effect of the treatments on renal inflammatory cytokine levels, including IL-1β, IL-6, and TNF-α. CP caused significant elevation of these pro-inflammatory markers compared to the control group. Riociguat at both doses (3 and 10 mg/kg) mitigated the CP-induced increases in cytokine levels. The reduction in IL-1β levels was more significant with the higher dose of riociguat (10 mg/kg) compared to the lower dose (3 mg/kg).

### 3.5. Effect on Kidney Morphology

**Figure 4:**
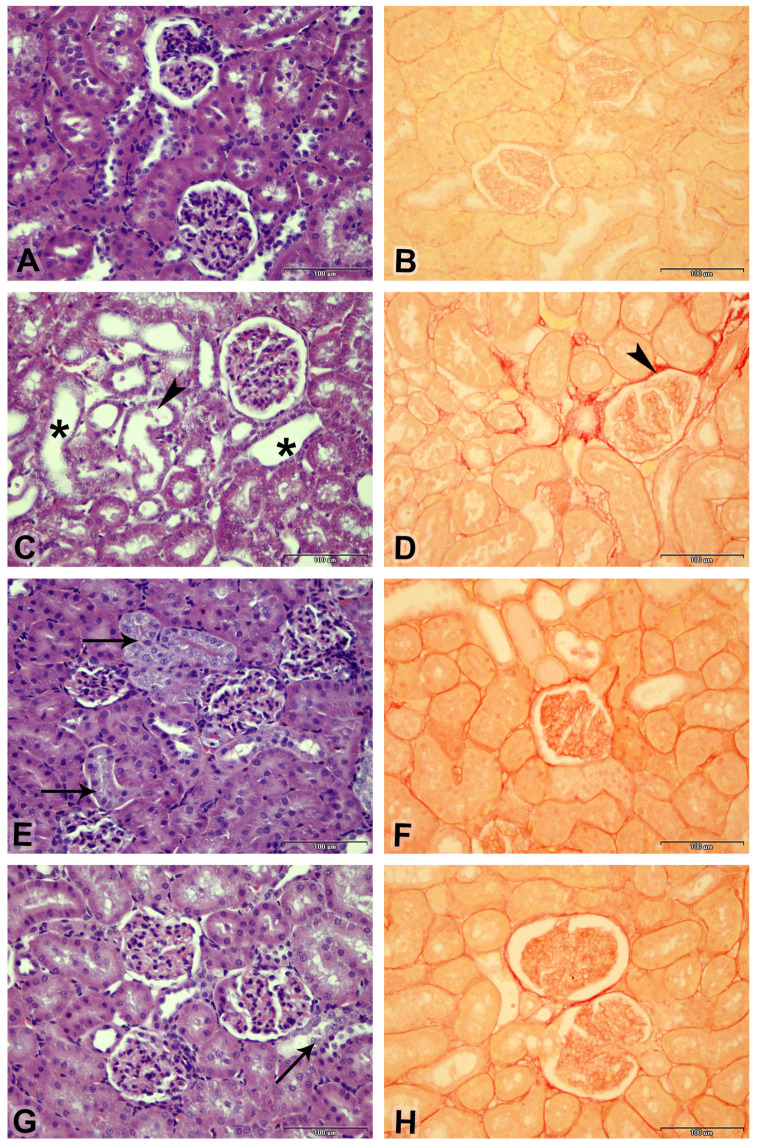
Photomicrographs of the renal cortex (scale bar = 100 µm). Slides (**A**,**C**,**E**,**G**) are stained with (**H**,**E**), while slides (**B**,**D**,**F**,**H**) are stained with Picro–Sirius Red: (**A**) Control group exhibits normal renal histological structures with intact glomerular tufts and renal tubules (Score 0). (**C**) Cisplatin (CP) treatment shows moderate cystic dilation of renal tubules (asterisk) and a few cellular casts (arrowheads) (Score 3). (**E**) CP + riociguat at 3 mg/kg (R3) displays basophilia in multiple renal tubules (arrow) with intact glomeruli (Score 2). (**G**) CP + riociguat at 10 mg/kg (R10) exhibits mild basophilia (arrow) in a renal tubule with intact glomeruli (Score 1). Slides (**B**,**D**,**F**,**H**) (Picro–Sirius Red staining) highlight red-stained collagen fibers (arrowheads) and yellow-stained non-collagenous structures across all groups.

Figure 4 illustrates the kidney architecture, showing that the control group exhibited normal renal structure. Treatment with PC resulted in significant histological changes characterized by cystic dilation, cellular casts, and fibrosis. Riociguat treatment at both doses alleviated CP-induced renal damage, leading to a significant reduction in acute tubular necrosis. Moreover, the fibrosis index and acute tubular necrosis, as shown in Table 3, were substantially higher in the CP-treated group compared to the control group. Riociguat administration at both doses effectively reversed these changes to a similar extent.

## 4. Discussion

Cis-diamminedichloroplatinum(II) (cisplatin, CP) is a commonly used chemotherapy drug for treating ovarian, bladder, lung, and testicular cancers [20]. Due to its effectiveness in inhibiting tumor growth, CP is prescribed to approximately 10–20% of cancer patients [21]; however, its clinical application is significantly restricted by nephrotoxicity, with acute kidney injury (AKI) affecting nearly 30% of patients undergoing cisplatin-based treatment [22]. Our study confirmed that a single intraperitoneal injection of CP at a dose of 6 mg/kg induces nephrotoxicity, supporting existing findings that CP accumulates in renal tissues due to its secretion and reabsorption in the renal tubules [23,24]. Proximal tubular epithelial cells accumulate CP and its metabolites at concentrations four to five times higher than in plasma [22], leading to severe cytotoxic effects, including inflammation, apoptosis, oxidative stress, and vascular injury [8,25].

CP-induced renal dysfunction is marked by a reduced glomerular filtration rate (GFR), resulting in proteinuria and elevated levels of renal function markers such as serum creatinine, urea, and uric acid [26]. Our findings are consistent with previous studies, demonstrating that CP treatment significantly increases urea, creatinine, uric acid, and NAGL, which are indicators of renal injury. These results align with reports showing that nephrotoxic agents, including CP, cause a significant decline in kidney function [22]. Histological analysis further confirmed CP-induced kidney damage, characterized by tubular vacuolization, leukocyte infiltration, epithelial necrosis, and glomerular basement membrane disruption—hallmarks of nephrotoxicity [27].

One of the primary mechanisms underlying CP-induced nephrotoxicity is oxidative stress, caused by excessive production of reactive oxygen species (ROS) during CP metabolism in renal tubular cells [28,29]. ROS trigger lipid peroxidation (LPO), leading to membrane damage and increased MDA levels, a byproduct of oxidative lipid degradation [30]. Our study found that CP significantly increased LPO markers while depleting the antioxidant defense system, including TAC, GR, SOD, and CAT. SOD plays a crucial role in converting highly toxic superoxide anions (O_2_^−^) into hydrogen peroxide (H_2_O_2_), which is subsequently detoxified by CAT [31]. The observed reduction in these antioxidant enzymes following CP treatment is consistent with previous research [32,33].

In our study, treatment with riociguat at doses of 3 and 10 mg/kg effectively mitigated CP-induced nephrotoxicity. Riociguat significantly reduced serum levels of urea, creatinine, uric acid, and NAGL, indicating improved kidney function. Additionally, riociguat enhanced antioxidant defense by increasing TAC, SOD, CAT, and GR activity while reducing LPO and MDA levels, suggesting its role as a potent free-radical scavenger [34]. These results are consistent with recent studies demonstrating that antioxidant therapy can alleviate CP-induced oxidative stress and renal damage [25].

The nephroprotective effects of riociguat observed in the present study are consistent with previous reports showing that sGC stimulators attenuate kidney injury by enhancing cGMP signaling, thereby reducing oxidative stress and inflammation [35]. Riociguat has been shown to improve renal function parameters, suppress pro-inflammatory cytokines, and preserve renal architecture in various models of acute kidney injury and chronic kidney disease [35,36]. In contrast, sGC activators may exert more pronounced antifibrotic and anti-apoptotic effects, particularly under conditions of chronic oxidative stress where NO bioavailability is reduced [37,38]. Unlike sGC stimulators, which require NO to activate sGC, activators directly target oxidized or heme-free sGC, potentially offering greater efficacy in disease states characterized by NO deficiency [39,40]. Both classes of sGC modulators, therefore, hold promise for nephroprotection, although their relative benefits may depend on the underlying pathophysiological context. Further comparative studies are warranted to define the optimal therapeutic applications of sGC stimulators versus activators in kidney disease [38,41].

Inflammation also plays a critical role in CP-induced nephrotoxicity as CP accumulation in renal tissue activates inflammatory cytokines such as IL-1β, IL-6, and TNF-α [42]. Several studies suggest that TNF-α is a key mediator of inflammatory responses following CP exposure, leading to elevated cytokine and chemokine levels [16]. Our study found that riociguat treatment significantly reduced IL-1β, IL-6, and TNF-α levels, confirming its anti-inflammatory properties. These findings suggest that riociguat may serve as a protective agent against CP-induced inflammation.

Histopathological analysis further demonstrated the nephroprotective effects of riociguat as it significantly reduced acute tubular necrosis, fibrosis index, and lesion scores compared to the CP-only group. The structural integrity of renal tubules and glomeruli was largely preserved in riociguat-treated groups, reinforcing its role in kidney tissue repair. These findings are in line with recent studies showing that pharmacological interventions targeting oxidative stress and inflammation can alleviate CP-induced renal damage [25].

**Figure 5:**
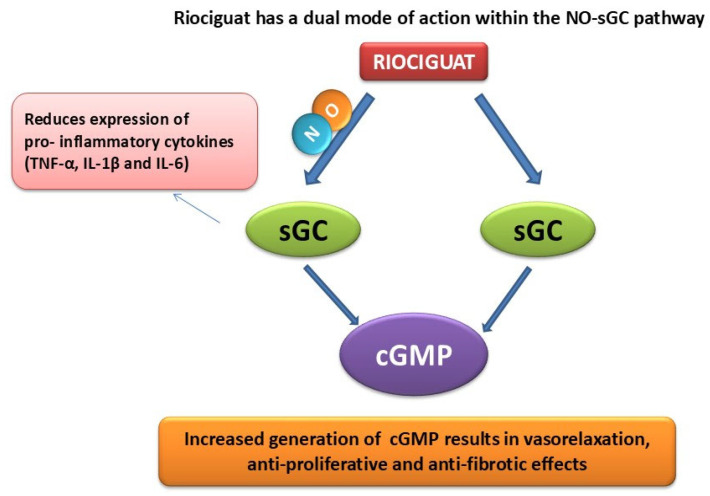
A summary of possible mechanisms related to renoprotection caused by riociguat.

Riociguat exerts its nephroprotective effects primarily through enhancement of the NO–sGC–cGMP signaling pathway. By stimulating sGC both independently and synergistically with NO, riociguat increases intracellular cGMP levels, leading to vasodilation and improved renal blood flow [43]. Elevated cGMP further mediates antifibrotic, antiproliferative, and anti-inflammatory actions by modulating signaling pathways that reduce oxidative stress and suppress the production of pro-inflammatory cytokines [44]. NO itself is a potent vasodilator that can act as either an inflammatory mediator or an anti-inflammatory agent, depending on the physiological context. Riociguat enhances NO signaling and bioavailability, thereby mitigating inflammation by neutralizing reactive oxygen species such as superoxide and downregulating adhesion molecules involved in leukocyte recruitment. In addition, the ability of riociguat to stabilize NO–sGC binding supports sustained cGMP production, even under conditions of oxidative stress that typically impair NO signaling [35]. Although these mechanisms are well established in the cardiovascular and pulmonary systems, their contribution to renal protection, particularly in acute kidney injury, is only beginning to be recognized and warrants further detailed investigation. Figure 5 summarizes the proposed mechanism of action of riociguat and its possible renal protective effect.

This study has limitations in that it did not investigate whether riociguat might affect the anticancer effect of cisplatin in a cancer model, offering more areas to explore in future studies.

## 5. Conclusions

Our findings indicate that riociguat has nephroprotective effects against CP-induced kidney injury by modulating renal function markers, reducing oxidative stress, and suppressing inflammatory cytokine activation. High-dose riociguat was more effective at restoring renal parameters, histopathological changes, and inflammation compared to the lower dose. Riociguat’s ability to restore antioxidant enzyme activity and protect renal tissue suggests its potential as an adjunct therapy in cisplatin-based chemotherapy. However, further clinical investigations are needed to validate these preclinical findings and explore riociguat’s potential for translation into clinical practice for cancer patients undergoing CP treatment.

## Figures and Tables

**Table 1:** Effect of riociguat on physiological changes and urine osmolality in cisplatin (CP)-caused acute kidney injury (AKI).

Parameters/Treatment	Control	CP	CP + R3	CP + R10
Baseline body weight (g)	282.0 ± 10.21	282.0 ± 9.55	281.3 ± 11.75	282.0 ± 10.01
Final body weight (g) taken on day 10	300.0 ± 8.80	268.8 ± 8.73	271.2 ± 9.90	278.3 ± 10.81
Body weight change (%)	6.55 ± 1.45	−4.59 ± 1.49 ^a^	−3.40 ± 1.41	−1.33 ± 0.97
Total kidney weight (g)	1.87 ± 0.08	1.96 ± 0.06	1.85 ± 0.05	1.67 ± 0.06 ^b^
Relative kidney weight (%)	0.62 ± 0.02	0.73 ± 0.02 ^a^	0.68 ± 0.02	0.60 ± 0.01 ^b^
Water intake (mL)	21.83 ± 0.79	46.50 ± 1.73 ^a^	40.67 ± 3.73	39.50 ± 2.08
Urine output (mL)	6.00 ± 0.73	41.67 ±1.05 ^a^	36.83 ± 2.71	33.33 ± 3.29 ^b^
Urine osmolality (mOsm/Kg)	2063.7 ± 179.9	510 ± 84.9 ^a^	789.3 ± 178.6	942.3 ± 70.1 ^b^

Values in the table are expressed as mean ± SEM (n = 6). Comparisons between groups were performed using one-way ANOVA, followed by Bonferroni’s post hoc test for multiple comparisons. A *p*-value of less than 0.05 was considered statistically significant. ^a^ denotes a statistically significant difference compared to the control group. ^b^ signifies a statistically significant difference relative to the CP group.

**Table 2:** Effect of two doses of riociguat (R) treatment on plasma parameters in rats with cisplatin (CP)-induced acute kidney injury (AKI).

CP + R10	CP + R3	CP	Control	Parameters/ Treatment
12.12 ± 1.36 ^b^	19.53 ± 3.06	20.06 ± 2.39 ^a^	3.58 ± 0.46	Urea (mmol/L)
85.3 ± 8.1 ^b^	116.9 ± 19.6 ^b^	127.6 ± 11.4 ^a^	23.2 ± 0.7	Creatinine (μmol/L)
24.53 ± 2.52	23.78 ± 4.04 ^b^	31.22 ± 2.27 ^a^	17.35 ± 1.43	Uric acid (μmol/L)
77.65 ± 3.00 ^b^	91.32 ± 7.45 ^b^	125.23 ± 4.56 ^a^	34.32 ± 3.26	NGAL (ng/mL)

Values in the table are presented as mean ± SEM (n = 6). NGAL: neutrophil gelatinase-associated lipocalin. Comparisons between groups were performed using one-way ANOVA, followed by Bonferroni’s post hoc test for multiple comparisons. A *p*-value of less than 0.05 was considered statistically significant. ^a^ denotes a statistically significant difference compared to the control group. ^b^ signifies a statistically significant difference relative to the CP group.

**Table 3:** Histopathological evaluation of kidney tissue following riociguat administration (3 mg/kg for R3 and 10 mg/kg for R10) in rats experiencing cisplatin (CP)-induced acute kidney injury (AKI).

Assessment/Treatment	Acute Tubular Necrosis		Fibrosis Index
	%	Lesion Score	%
Control	0.0 ± 0.0	0	5.1 ± 0.17
CP	27.8 ± 2.38 ^a^	3	12.05 ± 0.29 ^a^
CP + R3	19.4 ± 3.37 ^b^	2	9.24 ± 0.27 ^b^
CP + R10	7.2 ± 1.8 ^b^	1	8.07 ± 0.21 ^b^

Values in the table are expressed as mean ± SEM (n = 6). Comparisons between groups were performed using one-way ANOVA, followed by Bonferroni’s post hoc test for multiple comparisons. A *p*-value of less than 0.05 was considered statistically significant. ^a^ denotes a statistically significant difference compared to the control group. ^b^ signifies a statistically significant difference relative to the CP group.

## Data Availability

Data will be made available upon request.

## References

[1] Gameiro J., Fonseca J.A., Outerelo C., Lopes J.A. (2020). Acute Kidney Injury: From Diagnosis to Prevention and Treatment Strategies. J. Clin. Med..

[2] Verma S., Kellum J.A. (2021). Defining Acute Kidney Injury. Crit. Care Clin..

[3] Khwaja A. (2012). KDIGO Clinical Practice Guidelines for Acute Kidney Injury. Nephron Clin. Pract..

[4] Turgut F., Awad A.S., Abdel-Rahman E.M. (2023). Acute Kidney Injury: Medical Causes and Pathogenesis. J. Clin. Med..

[5] Tomsa A.M., Alexa A.L., Junie M.L., Rachisan A.L., Ciumarnean L. (2019). Oxidative Stress as a Potential Target in Acute Kidney Injury. PeerJ.

[6] Saleh S., Becker C., Frey R., Mück W. (2016). Population Pharmacokinetics of Single-Dose Riociguat in Patients with Renal or Hepatic Impairment. Pulm. Circ..

[7] Ali B.H., Al-Salam S., Al Husseini I.S., Al-Lawati I., Waly M., Yasin J., Fahim M., Nemmar A. (2013). Abrogation of Cisplatin-Induced Nephrotoxicity by Emodin in Rats. Fundam. Clin. Pharmacol..

[8] McSweeney K.R., Gadanec L.K., Qaradakhi T., Ali B.A., Zulli A., Apostolopoulos V. (2021). Mechanisms of Cisplatin-Induced Acute Kidney Injury: Pathological Mechanisms, Pharmacological Interventions, and Genetic Mitigations. Cancers.

[9] Elmorsy E.A., Saber S., Hamad R.S., Abdel-Reheim M.A., El-Kott A.F., AlShehri M.A., Morsy K., Salama S.A., Youssef M.E. (2024). Advances in Understanding Cisplatin-Induced Toxicity: Molecular Mechanisms and Protective Strategies. Eur. J. Pharm. Sci..

[10] Santos M.L.C., de Brito B.B., da Silva F.A.F., Botelho A.C.D.S., de Melo F.F. (2020). Nephrotoxicity in Cancer Treatment: An Overview. World J. Clin. Oncol..

[11] Chen C., Xie D., Gewirtz D.A., Li N. (2022). Nephrotoxicity in Cancer Treatment: An Update. Adv. Cancer Res..

[12] Perše M., Večerić-Haler Ž. (2018). Cisplatin-Induced Rodent Model of Kidney Injury: Characteristics and Challenges. BioMed Res. Int..

[13] Conole D., Scott L.J. (2013). Riociguat: First Global Approval. Drugs.

[14] Kenny M., Clarke M.M., Pogue K.T. (2022). Overview of Riociguat and Its Role in the Treatment of Pulmonary Hypertension. J. Pharm. Pract..

[15] Frey R., Becker C., Saleh S., Unger S., van der Mey D., Mück W. (2018). Clinical Pharmacokinetic and Pharmacodynamic Profile of Riociguat. Clin. Pharmacokinet..

[16] Al Suleimani Y.M., Ali B.H., Ali H., Manoj P., Almashaiki K.S., Abdelrahman A.M. (2024). The Salutary Effects of Diminazene, Lisinopril or Valsartan on Cisplatin-Induced Acute Kidney Injury in Rats: A Comparative Study. Physiol. Res..

[17] Geschka S., Kretschmer A., Sharkovska Y., Evgenov O.V., Lawrenz B., Hucke A., Hocher B., Stasch J.P. (2011). Soluble Guanylate Cyclase Stimulation Prevents Fibrotic Tissue Remodeling and Improves Survival in Salt-Sensitive Dahl Rats. PLoS ONE.

[18] Upreti G.C., Davis C., Oliver J. (1991). Preparation of Representative Homogenates of Biological Tissues: Effect of Salt on Protein Extraction. Anal. Biochem..

[19] Ali B.H., Al-Salam S., Al Za’abi M., Al Balushi K.A., Ramkumar A., Waly M.I., Yasin J., Adham S.A., Nemmar A. (2014). Does Swimming Exercise Affect Experimental Chronic Kidney Disease in Rats Treated with Gum Acacia?. PLoS ONE.

[20] Volarevic V., Djokovic B., Jankovic M.G., Harrell C.R., Fellabaum C., Djonov V., Arsenijevic N. (2019). Molecular Mechanisms of Cisplatin-Induced Nephrotoxicity. J. Biomed. Sci..

[21] Pabla N., Dong Z. (2008). Cisplatin Nephrotoxicity: Mechanisms and Renoprotective Strategies. Kidney Int..

[22] Miller R.P., Tadagavadi R.K., Ramesh G., Reeves W.B. (2010). Mechanisms of Cisplatin Nephrotoxicity. Toxins.

[23] Abdelrahman A.M., Al Suleimani Y., Shalaby A., Ashique M., Manoj P., Al-Saadi H., Ali B.H. (2019). Effect of Levosimendan, a Calcium Sensitizer, on Cisplatin-Induced Nephrotoxicity in Rats. Toxicol. Rep..

[24] Imam F., Kothiyal P., Alshehri S., Afzal M., Iqbal M., Khan M.R., Alanazi A.A.H., Anwer M.K. (2023). Hirsutidin Prevents Cisplatin-Evoked Renal Toxicity by Reducing Oxidative Stress/Inflammation and Restoring the Endogenous Enzymatic and Non-Enzymatic Level. Biomedicines.

[25] Anwer T., Alshahrani S., Somaili A.M.H., Khubrani A.H., Ahmed R.A., Jali A.M., Alshamrani A., Rashid H., Nomeir Y., Khalid M. (2023). Nephroprotective Effect of Diosmin against Cisplatin-Induced Kidney Damage by Modulating IL-1β, IL-6, TNFα and Renal Oxidative Damage. Molecules.

[26] Cetin R., Devrim E., Kiliçoğlu B., Avci A., Candir O., Durak I. (2006). Cisplatin Impairs Antioxidant System and Causes Oxidation in Rat Kidney Tissues: Possible Protective Roles of Natural Antioxidant Foods. J. Appl. Toxicol..

[27] Gyurászová M., Gurecká R., Bábíčková J., Tóthová Ľ. (2020). Oxidative Stress in the Pathophysiology of Kidney Disease: Implications for Noninvasive Monitoring and Identification of Biomarkers. Oxid. Med. Cell. Longev..

[28] Baud L., Ardaillou R. (1986). Reactive Oxygen Species: Production and Role in the Kidney. Am. J. Physiol..

[29] Ma N., Wei W., Fan X., Ci X. (2019). Farrerol Attenuates Cisplatin-Induced Nephrotoxicity by Inhibiting the Reactive Oxygen Species-Mediated Oxidation, Inflammation, and Apoptotic Signaling Pathways. Front. Physiol..

[30] Su L.J., Zhang J.H., Gomez H., Murugan R., Hong X., Xu D., Jiang F., Peng Z.Y. (2019). Reactive Oxygen Species-Induced Lipid Peroxidation in Apoptosis, Autophagy, and Ferroptosis. Oxid. Med. Cell. Longev..

[31] Couto N., Wood J., Barber J. (2016). The Role of Glutathione Reductase and Related Enzymes on Cellular Redox Homoeostasis Network. Free Radic. Biol. Med..

[32] Bibi Sadeer N., Montesano D., Albrizio S., Zengin G., Mahomoodally M.F. (2020). The Versatility of Antioxidant Assays in Food Science and Safety—Chemistry, Applications, Strengths, and Limitations. Antioxidants.

[33] Vašková J., Kočan L., Vaško L., Perjési P. (2023). Glutathione-Related Enzymes and Proteins: A Review. Molecules.

[34] Safhi M.M., Qumayri H.M., Masmali A.U.M., Siddiqui R., Alam M.F., Khan G., Anwer T. (2019). Thymoquinone and Fluoxetine Alleviate Depression via Attenuating Oxidative Damage and Inflammatory Markers in Type-2 Diabetic Rats. Arch. Physiol. Biochem..

[35] Al-Maskari R., Abdelrahman A.M., Ali H., Manoj P., Al Suleimani Y. (2024). Nephroprotective Effects of the Soluble Guanylyl Cyclase Stimulator, Riociguat, in Doxorubicin-Induced Acute Kidney Injury in Rats. Toxicol. Rep..

[36] Sravani S., Saifi M.A., Godugu C. (2020). Riociguat Ameliorates Kidney Injury and Fibrosis in an Animal Model. Biochem. Biophys. Res. Commun..

[37] Chen X., Xiong Y., Zeng S., Delić D., Gaballa M., Kalk P., Klein T., Krämer B.K., Hocher B. (2024). Comparison of sGC Activator and sGC Stimulator in 5/6 Nephrectomized Rats on High-Salt Diet. Front. Pharmacol..

[38] Balzer M.S., Pavkovic M., Frederick J., Abedini A., Freyberger A., Vienenkötter J., Mathar I., Siudak K., Eitner F., Sandner P. (2023). Treatment Effects of Soluble Guanylate Cyclase Modulation on Diabetic Kidney Disease at Single-Cell Resolution. Cell Rep. Med..

[39] Stasch J.P., Schlossmann J., Hocher B. (2015). Renal Effects of Soluble Guanylate Cyclase Stimulators and Activators: A Review of the Preclinical Evidence. Curr. Opin. Pharmacol..

[40] Sandner P., Follmann M., Becker-Pelster E., Hahn M.G., Meier C., Freitas C., Roessig L., Stasch J.P. (2024). Soluble GC Stimulators and Activators: Past, Present and Future. Br. J. Pharmacol..

[41] Sharma N., Liu W., Tsai X.Q., Wang Z., Outtrim C., Tang A., Pieper M.P., Reinhart G.A., Huang Y. (2025). A Novel Soluble Guanylate Cyclase Activator, Avenciguat, in Combination with Empagliflozin, Protects against Renal and Hepatic Injury in Diabetic db/db Mice. Am. J. Physiol. Endocrinol. Metab..

[42] Ramesh G., Reeves W.B. (2006). Cisplatin Increases TNF-Alpha mRNA Stability in Kidney Proximal Tubule Cells. Ren. Fail..

[43] Ghofrani H.A., Humbert M., Langleben D., Schermuly R., Stasch J.P., Wilkins M.R., Klinger J.R. (2017). Riociguat: Mode of action and clinical development in pulmonary hypertension. Chest.

[44] Schwabl P., Brusilovskaya K., Supper P., Bauer D., Königshofer P., Riedl F., Hayden H., Fuchs C.D., Stift J., Oberhuber G. (2018). The soluble guanylate cyclase stimulator riociguat reduces fibrogenesis and portal pressure in cirrhotic rats. Sci. Rep..

